# Chromosome 1p36 candidate gene ZNF436 predicts the prognosis of neuroblastoma: a bioinformatic analysis

**DOI:** 10.1186/s13052-023-01549-x

**Published:** 2023-10-31

**Authors:** Haiwei Wang, Xinrui Wang, Liangpu Xu

**Affiliations:** Fujian Maternity and Child Health Hospital, Affiliated Hospital of Fujian Medical University, Fuzhou, Fujian China

**Keywords:** Neuroblastoma, 1p deletion, MYCN amplification, ZNF436, Nomogram model

## Abstract

**Background:**

Genetic 1p deletion is reported in 30% of all neuroblastoma and is associated with the unfavorable prognosis of neuroblastoma. The expressions and prognosis of 1p candidate genes in neuroblastoma are unclear.

**Methods:**

Public neuroblastoma cohorts were obtained for secondary analysis. The prognosis of 1p candidate genes in neuroblastoma was determined using Kaplan-Meier and cox regression analysis. The prediction of the nomogram model was determined using timeROC.

**Results:**

First, we confirmed the bad prognosis of 1p deletion in neuroblastoma. Moreover, zinc finger protein 436 (ZNF436) located at 1p36 region was down-regulated in 1p deleted neuroblastoma and higher ZNF436 expression was associated with the longer event free survival and overall survival of neuroblastoma. The expression levels of ZNF436 were lower in neuroblastoma patients with MYCN amplification or age at diagnosis ≥ 18months, or with stage 4 neuroblastoma. ZNF436 had robust predictive values of MYCN amplification and overall survival of neuroblastoma. Furthermore, the prognostic significance of ZNF436 in neuroblastoma was independent of MYCN amplification and age of diagnosis. Combinations of ZNF436 with MYCN amplification or age of diagnosis achieved better prognosis. At last, we constructed a nomogram risk model based on age, MYCN amplification and ZNF436. The nomogram model could predict the overall survival of neuroblastoma with high specificity and sensitivity.

**Conclusions:**

Chromosome 1p36 candidate gene ZNF436 was a prognostic maker of neuroblastoma.

## Background

Neuroblastoma is a common extracranial tumor in children [1, 2]. The clinical outcomes of high risk neuroblastoma are unsatisfied and about 10-15% of pediatric cancer related mortality is associated with neuroblastoma [3, 4]. Neuroblastoma is a heterogeneous disease. The malignancy and clinical outcomes of neuroblastoma are significantly varied [5]. Known prognostic factors of neuroblastoma include age at diagnosis, neuroblastoma stage and MYCN amplification [6]. MYCN maps to the chromosome 2p24.3 region and gain of MYCN copy number variation is detected in about 25% high risk neuroblastoma [3]. The size of MYCN amplicon ranges from 100 to 1500 Kb, including MYCN, DDX1 and some other genes [7, 8]. Except 2p24.3 amplification, overall survival is lower in neuroblastoma patients harboring telomere, RAS and TP53 mutations [9]. Understanding the correlations between genetic aberrations and clinical features of neuroblastoma will provide novel therapeutic targets and prognostic makers for neuroblastoma.

Loss of 1p, 11q and gain of 17q amplification are also well known biomarkers correlated with the clinical risks and outcomes of neuroblastoma [10–12]. Particularly, deletion of 1p36 has been reported in 10-30% of neuroblastoma and is associated with the unfavorable prognosis of neuroblastoma [13, 14]. Chromosome 1p36 regions harbor multiple potential neuroblastoma suppressor genes [15, 16]. For example, CHD5 is located at 1p36.31 region [17, 18] and is associated with the lower overall survival of neuroblastoma [19, 20]. Furthermore, forced expression of CHD5 in neuroblastoma cells with 1p deletion suppresses the metastatic progress of neuroblastoma [21]. TP73 is a TP53 homologue and localized at 1p36.3 region [22]. TP73 could regulate the expressions of MYCN [23] and induce the differentiation of neuroblastoma [24]. Chromosome 1p36.22 candidate gene KIF1B is a tumor suppressor gene in neuroblastoma [25] and germline KIF1B deletion is associated with high predisposition of neuroblastoma development [26]. All those results highlighted the important pathogenic roles of 1p candidate genes in neuroblastoma. However, the expressions and prognosis of neuroblastoma suppressor genes in chromosome 1p regions are not studied in a comprehensive manner.

Using published neuroblastoma cohorts, we analyzed the expressions and prognosis of 1p candidate genes. Our results suggested that zinc finger protein 436 (ZNF436) was an independent prognostic marker and was significantly associated with the favorable clinical outcomes of neuroblastoma. Combinations of ZNF436, MYCN amplification or age at diagnosis achieved better prognosis in neuroblastoma. Finally, we showed that a nomogram risk model based on age, MYCN amplification and ZNF436 could predict the overall survival of neuroblastoma with high specificity and sensitivity.

## Methods

### Data collection

Therapeutically Applicable Research to Generate Effective Treatments (TARGET) datasets were collected from https://ocg.cancer.gov/ website [27]. The copy number variations of TARGET dataset were downloaded from cBioPortal (http://www.cbioportal.org/) [28, 29]. E-MTAB-1781 dataset [30] was collected from The European Molecular Biology Laboratory-European Bioinformatics Institute (EMBL-EBI) (https://www.ebi.ac.uk/arrayexpress/). The Gene Expression Omnibus (GEO) datasets GSE13136 [31], GSE73517 [32], GSE16476 [33–35], GSE62564 [36] and GSE85047 [37] were collected from www.ncbi.nlm.nih.gov/geo website. All datasets were analyzed using R software. The expression of genes in neuroblastoma were showed using “pheatmap” package. All the samples used in this study were derived from primary untreated neuroblastoma tumors.

### Univariate and multivariate cox regression analysis

Packages “survival” and “survminer” were used for univariate and multivariate cox regression analysis. Packages “forestplot” and “ggforest” were used to generate the forest plots. The Hazard ratio (HR) and P values were determined during cox regression survival analysis.

### Kaplan-Meier survival analysis

Kaplan-Meier survival analysis was carried out using “survival” and “survminer” packages. Neuroblastoma patients were classified into “high” or “low” groups by the best cutoff points. P values were determined by log-rank test.

### Receiver operating characteristic (ROC) and timeROC analysis

The ROC curves were plotted by ‘pROC’ package. The timeROC curves were generated using “timeROC” package. The area under the ROC curve (AUC) was determined by the ‘pROC’ and “survival” packages.

### Construction and validation of nomogram model

First, nomogram models were constructed using packages“rms” and “ggplot2” based on age, MYCN amplification and ZNF436 expression levels. Second, the accuracy of the nomogram model was further evaluated using calibration diagram. Third, the risk point of each patient in the nomogram model was determined by “nomogramFormula”. Neuroblastoma patients were classified into higher risk groups or lower risk groups. At last, Kaplan-Meier survival analysis and timeROC analysis were used to validate the prognostic roles of the nomogram models.

### Statistical analysis

The statistical P values were performed using two tails paired student’s t test. P values less than 0.05 were indicated significant difference.

## Results

### Genetic 1p deletion is an Independent prognostic marker of neuroblastoma

**Fig. 1:**
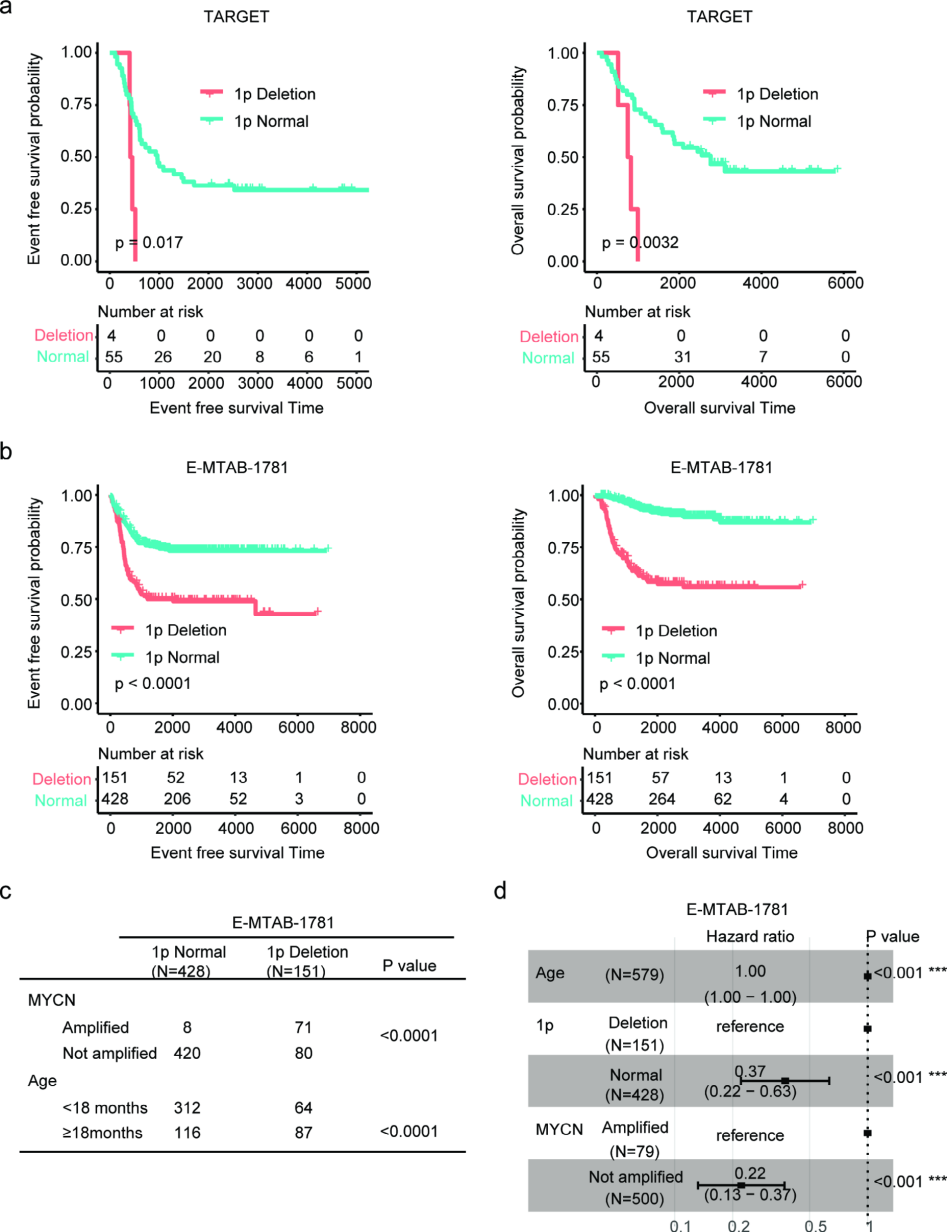
Prognosis of 1p deletion in neuroblastoma. (**a**) Event free survival and overall survival of neuroblastoma with 1p deletion or without 1p deletion in TARGET dataset. P values were determined by Log-rank test. (**b**) Event free survival and overall survival of neuroblastoma with 1p deletion or without 1p deletion in E-MTAB-1781 dataset. (**c**) Clinical characteristics of neuroblastoma patients in E-MTAB-1781 dataset. (**d**) Forest plot showed the prognosis of age, MYCN amplification and 1p deletion in E-MTAB-1781 dataset. Hazard ratio (HR) and P values were determined by multivariate cox regression analysis

First, we confirmed the prognosis of 1p deletion in neuroblastoma using TARGET copy number variation dataset. The copy number variation dataset in TARGET only included 59 neuroblastoma patients and only four neuroblastoma patients were with 1p36 deletion. Compared with neuroblastoma patients with normal 1p36, neuroblastoma patients with 1p36 deletion had shorter event free survival (Fig. 1a). Moreover, neuroblastoma patients carrying 1p36 deletion had worse overall survival, contrast with neuroblastoma patients with intact 1p36 in TARGET dataset (Fig. 1a).

The prognosis of 1p deletion was further confirmed using E-MTAB-1781 dataset. There were 151 neuroblastoma patients with 1p deletion, while, 428 neuroblastoma patients were with normal 1p. Similarly, 1p deletion was correlated with the prognosis of neuroblastoma in E-MTAB-1781 dataset. Compared with neuroblastoma patients with normal 1p, neuroblastoma patients with 1p deletion had shorter event free survival and overall survival (Fig. 1b).

Age at diagnosis and MYCN amplification are critical prognostic makers of neuroblastoma [38]. More than 98% neuroblastoma patients with normal 1p were without MYCN amplification, while, 47% neuroblastoma patients with 1p deletion were with amplified MYCN (Fig. 1c). Moreover, neuroblastoma patients with age at diagnosis < 18 months were associated with 1p intact status, while neuroblastoma patients with age at diagnosis ≥ 18 months were associated with 1p deletion status (Fig. 1c). Furthermore, 1p deletion was a prognostic factor of neuroblastoma in E-MTAB-1781, independent of MYCN amplification and age at diagnosis (Fig. 1d).

### Expressions and prognosis of 1p candidate genes in neuroblastoma

**Fig. 2:**
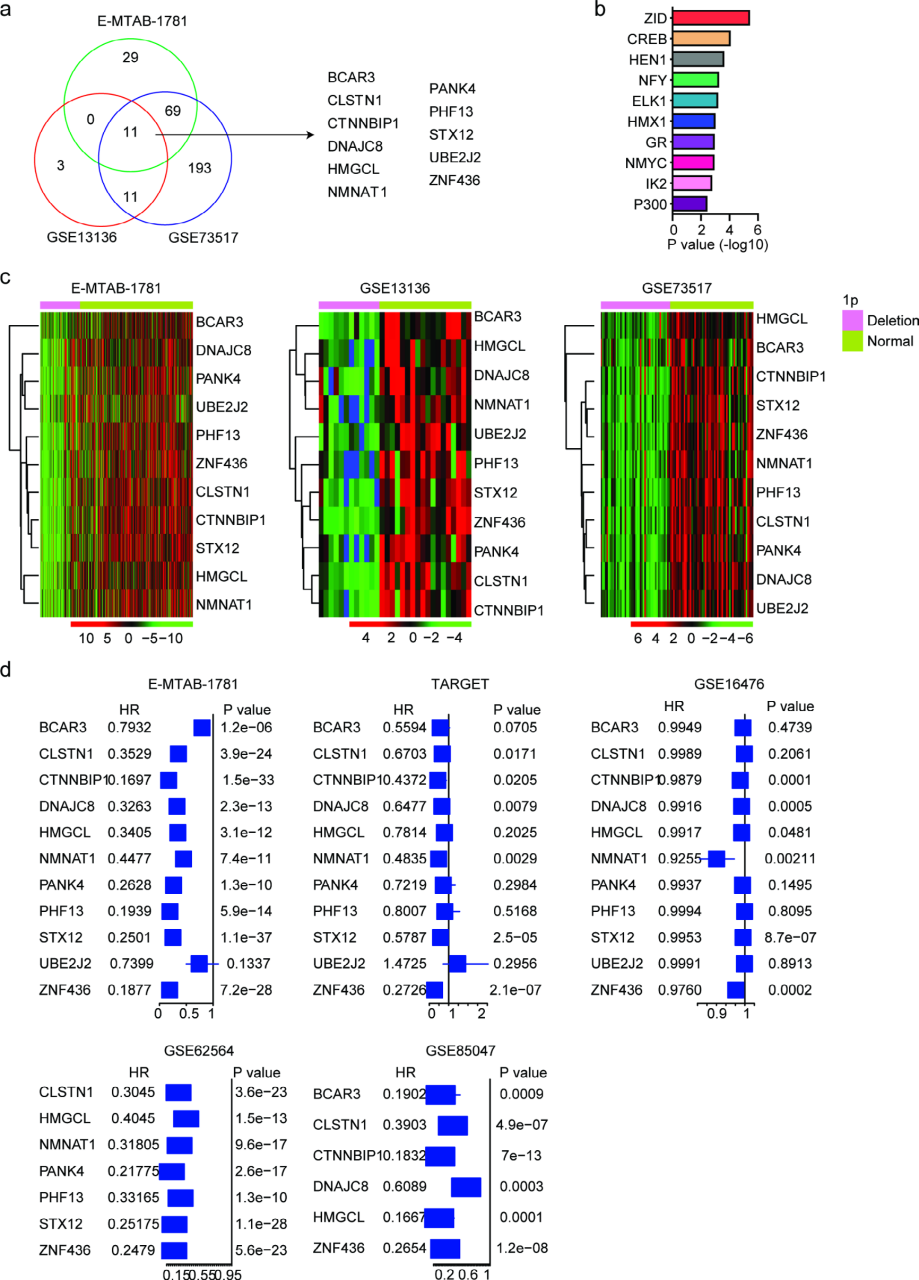
Expressions and prognosis of 1p candidate genes in neuroblastoma. (**a**) The common differentially expressed genes in 1p deleted neuroblastoma in E-MTAB-1781, GSE13136 and GSE73517 datasets. (**b**) Enriched transcription factors using the differentially expressed genes in 1p deleted neuroblastoma in E-MTAB-1781 dataset. (**c**) Expressions of BCAR3, CLSTN1, CTNNBIP1, DNAJC8, HMGCL, NMNAT1, PANK4, PHF13, STX12, UBE2J2 and ZNF436 in neuroblastoma with 1p deletion or without 1p deletion in E-MTAB-1781, GSE13136 and GSE73517 datasets. (**d**) Forest plots showed the associations of BCAR3, CLSTN1, CTNNBIP1, DNAJC8, HMGCL, NMNAT1, PANK4, PHF13, STX12, UBE2J2 and ZNF436 with the neuroblastoma overall survival in E-MTAB-1781, TARGET, GSE16476, GSE62564 and GSE85047 datasets. HR and P values were determined by univariate cox regression analysis

Genetic 1p regions include multiple tumor suppressor genes. However, not all genes located in 1p were down-regulated with the deletion of 1p and associated with the prognosis of neuroblastoma. So, we tried to analyze the expressions and prognosis of 1p candidate genes in neuroblastoma cohorts. Based on TARGET dataset in cBioPortal, 773 genes in chromosomal 1p regions were deleted in neuroblastoma patients. Compared with 1p normal neuroblastoma patients, only 105 genes in 1p regions were differentially expressed in 1p deleted neuroblastoma patients in E-MTAB-1781 dataset (Fig. 2a). Interestingly, those differentially expressed genes in 1p regions were associated with MYCN transcription factor (Fig. 2b).

The differentially expressed genes in 1p deleted neuroblastoma patients in GSE13136 and GSE73517 datasets were also identified. There were 25 and 280 genes in 1p regions were differentially expressed in 1p deleted patients in GSE13136 and GSE73517 datasets, respectively (Fig. 2a). Among them, BCAR3, CLSTN1, CTNNBIP1, DNAJC8, HMGCL, NMNAT1, PANK4, PHF13, STX12, UBE2J2 and ZNF436 were commonly changed in 1p deleted neuroblastoma patients in E-MTAB-1781, GSE13136 and GSE73517 datasets (Fig. 2a). And all those genes were down-regulated in 1p deleted neuroblastoma patients (Fig. 2c).

Furthermore, we determined the prognostic effects of those commonly down-regulated genes in neuroblastoma patients using univariate cox regression analysis. In E-MTAB-1781 dataset, BCAR3, CLSTN1, CTNNBIP1, DNAJC8, HMGCL, NMNAT1, PANK4, PHF13, STX12 and ZNF436 were all associated with the favorable prognosis of neuroblastoma. However, UBE2J2 had not prognosis of neuroblastoma (Fig. 2d). The prognostic effects of BCAR3, CLSTN1, CTNNBIP1, DNAJC8, HMGCL, NMNAT1, PANK4, PHF13, STX12, UBE2J2 and ZNF436 in neuroblastoma patients were further validated using TARGET, GSE16476, GSE62564 and GSE85047 datasets. CTNNBIP1, DNAJC8, NMNAT1, STX12 and ZNF436 represented significantly prognostic factors in both TARGET and GSE16476 datasets (Fig. 2d). Only a few differentially expressed genes were detected in GSE62564 and GSE85047 datasets and all those detected genes were associated with the favorable prognosis of neuroblastoma (Fig. 2d). Results from five independent neuroblastoma cohorts suggested that ZNF436 was detected and was associated with the favorable prognosis of neuroblastoma in E-MTAB-1781, TARGET, GSE16476, GSE62564 and GSE85047 datasets.

### High expression levels of ZNF436 are associated with the favorable prognosis of neuroblastoma

**Fig. 3:**
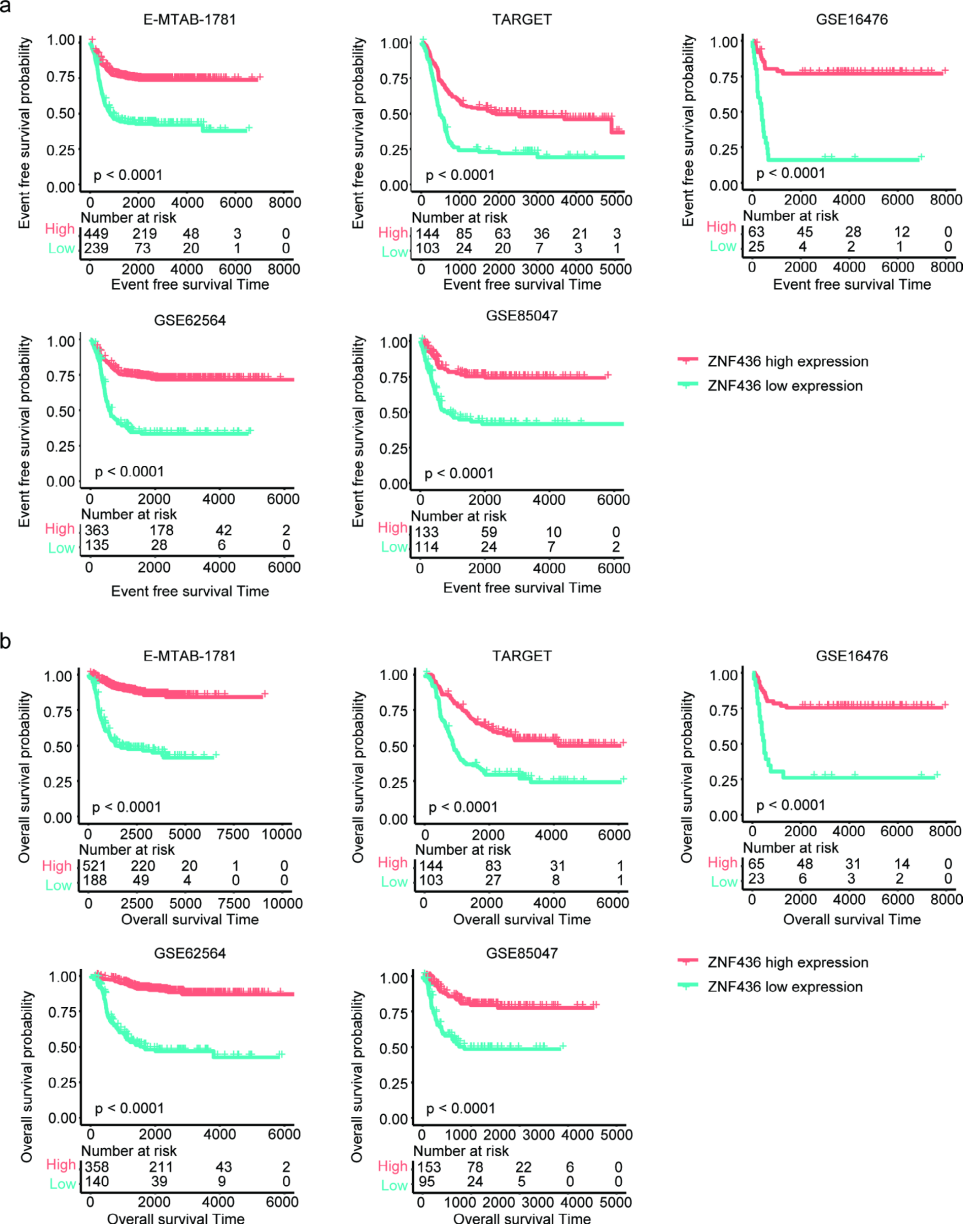
High expression levels of ZNF436 are associated with the favorable prognosis of neuroblastoma. (**a**) The Kaplan-Meier curves showed the different event free survival of neuroblastoma patients with ZNF436 higher expressions or lower expressions in E-MTAB-1781, TARGET, GSE16476, GSE62564 and GSE85047 datasets. (**b**) Different overall survival of neuroblastoma patients with ZNF436 higher expressions or lower expressions in E-MTAB-1781, TARGET, GSE16476, GSE62564 and GSE85047 datasets

ZNF436 is a transcription factor in 1p36.12 regions. The Kaplan-Meier survival analysis was further used to validate the favorable prognosis of ZNF436 in neuroblastoma. Neuroblastoma patients with ZNF436 higher expression levels had prolonged event free survival contrast with ZNF436 lowly expressed neuroblastoma patients in E-MTAB-1781 dataset (Fig. 3a). Also, neuroblastoma patients with ZNF436 higher expression levels had prolonged overall survival contrast with ZNF436 lowly expressed neuroblastoma patients in E-MTAB-1781 dataset (Fig. 3b).

The prognostic effects of ZNF436 in neuroblastoma were further validated using TARGET, GSE16476, GSE62564 and GSE85047 datasets. Similar with E-MTAB-1781 dataset, neuroblastoma patients with ZNF436 higher expression levels had prolonged event free survival (Fig. 3a) and overall survival (Fig. 3b), contrast with ZNF436 lowly expressed neuroblastoma patients in TARGET, GSE16476, GSE62564 and GSE85047 datasets.

### Expressions of ZNF436 in different sub-types of neuroblastoma

**Fig. 4:**
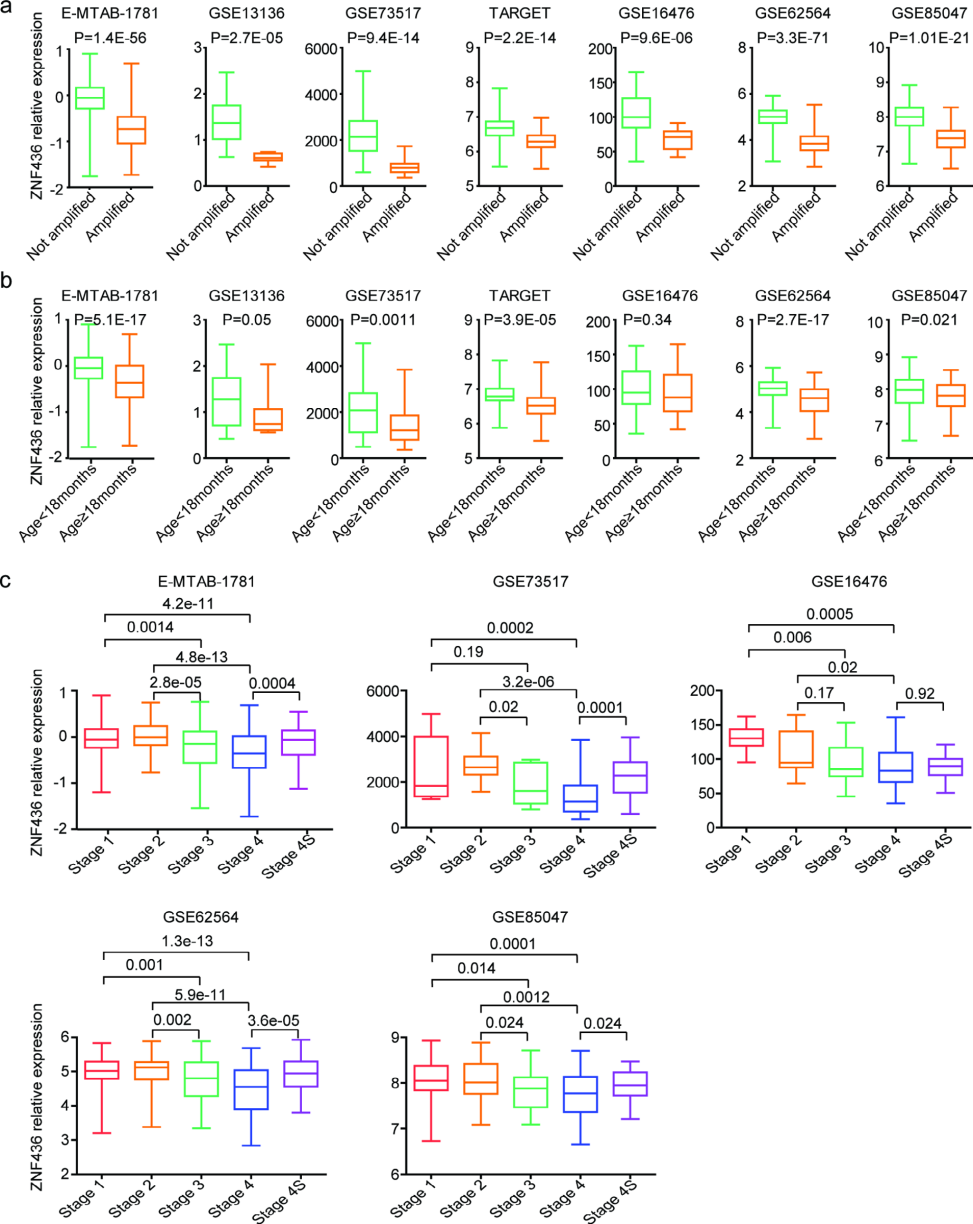
Expressions of ZNF436 in different sub-types of neuroblastoma. (**a**) Box plots showed the relative ZNF436 expression levels in neuroblastoma patients with or without MYCN amplification in E-MTAB-1781, GSE13136, GSE73517, TARGET, GSE16476, GSE62564 and GSE85047 datasets. P values were performed using Student’s t test. (**b**) The relative ZNF436 expression levels in neuroblastoma patients with age of diagnosis ≥ 18month or < 18months in E-MTAB-1781, GSE13136, GSE73517, TARGET, GSE16476, GSE62564 and GSE85047 datasets. (**c**) The relative ZNF436 expression levels in neuroblastoma patients with different tumor stages in E-MTAB-1781, GSE73517, GSE16476, GSE62564 and GSE85047 datasets

Age at diagnosis, MYCN amplification and neuroblastoma stage are critical biomarkers of neuroblastoma [38]. So, we determined the ZNF436 expressions in different sub-types of neuroblastoma using seven independent neuroblastoma cohorts. ZNF436 was down-regulated in MYCN amplified neuroblastoma patients in E-MTAB-1781, GSE13136, GSE73517, TARGET, GSE16476, GSE62564 and GSE85047 datasets (Fig. 4a). Also, in E-MTAB-1781, GSE13136, GSE73517, TARGET, GSE16476, GSE62564 and GSE85047 datasets, the expression levels of ZNF436 were lower in neuroblastoma patients with age at diagnosis ≥ 18months than neuroblastoma patients with age at diagnosis < 18months (Fig. 4b).

Moreover, contrast with stage 1 and stage 2 neuroblastoma, ZNF436 was down-regulated in stage 3 neuroblastoma patients in E-MTAB-1781, TARGET, GSE16476, GSE62564 and GSE85047 datasets (Fig. 4c). Also, contrast with stage 1 and stage 2 neuroblastoma, ZNF436 was down-regulated in stage 4 neuroblastoma patients in E-MTAB-1781, GSE13136, GSE73517, TARGET, GSE16476, GSE62564 and GSE85047 datasets (Fig. 4c). However, compared with stage 4, ZNF436 expressions in stage 4s neuroblastoma in E-MTAB-1781, GSE13136, GSE73517, TARGET, GSE16476, GSE62564 and GSE85047 datasets were not significantly different (Fig. 4c).

### ZNF436 is a predictor of the sub-types and overall survival of neuroblastoma

**Fig. 5:**
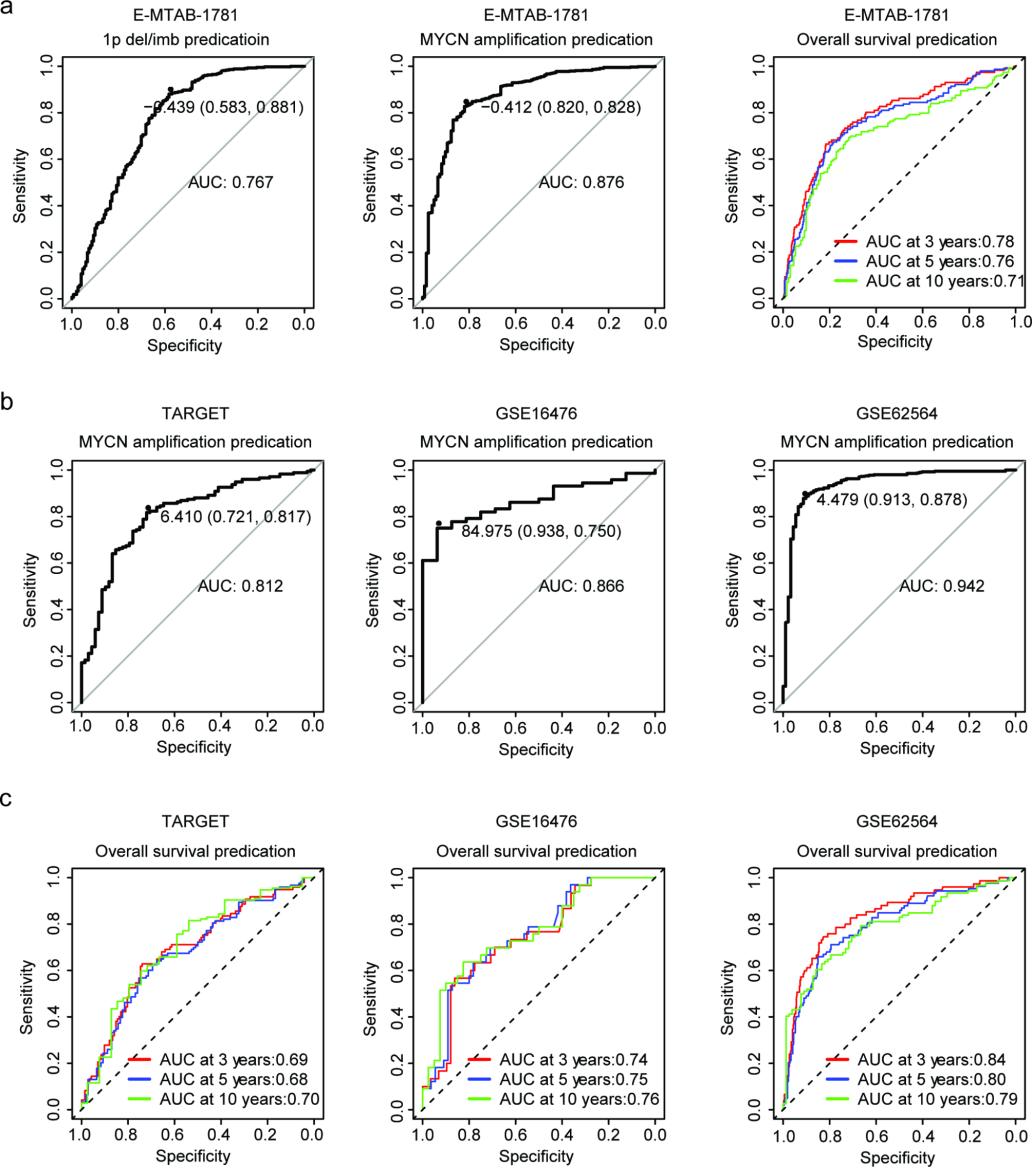
ZNF436 is a predictor of the sub-types and overall survival of neuroblastoma. (**a**) The ROC curves showed the specificity and sensitivity of ZNF436 in the prediction of 1p deletion, MYCN amplification and in the prediction of the overall survival of neuroblastoma in E-MTAB-1781 dataset. (**b**) Prediction of MYCN amplification by ZNF436 in TARGET, GSE16476 and GSE62564 datasets. (**c**) Prediction of the overall survival of neuroblastoma by ZNF436 in TARGET, GSE16476 and GSE62564 datasets

Our results suggested that ZNF436 was down-regulated with genetic 1p deletion or MYCN amplification and served as a favorable prognostic marker of neuroblastoma. Further, we attempted to determine the accuracy of ZNF436 in the prediction of 1p deletion, MYCN amplification and in the prediction of the overall survival of neuroblastoma. The ROC analysis in E-MTAB-1781 dataset indicated that ZNF436 could distinguish 1p deleted from 1p normal neuroblastoma patients with high specificity and sensitivity (Fig. 5a). Similar predictive specificity and sensitivity of ZNF436 in distinguishing MYCN amplified from MYCN non-amplified neuroblastoma patients was determined in E-MTAB-1781 dataset (Fig. 5a). Furthermore, ZNF436 also could predict the the three years, five years or ten years overall survival of neuroblastoma with high accuracy in E-MTAB-1781 dataset (Fig. 5a).

The predictions of MYCN amplification and overall survival of neuroblastoma based on ZNF436 expression were validated in TARGET, GSE16476 and GSE62564 datasets. Similarly, ZNF436 had robust predictive values of MYCN amplification. In TARGET, GSE16476 and GSE62564 neuroblastoma cohorts, ROC curves showed the high specificity and sensitivity of ZNF436 in distinguishing MYCN amplified from MYCN non-amplified neuroblastoma patients (Fig. 5b). Furthermore, ZNF436 also could predict the three years, five years or ten years overall survival of neuroblastoma with high accuracy in GSE16476 and GSE62564 datasets (Fig. 5c). However, prediction of overall survival of neuroblastoma in TARGET dataset was not accurate (Fig. 5c). All those results highlighted the sub-types and overall survival predictions of ZNF436 in neuroblastoma.

### ZNF436 is an Independent prognostic factor of neuroblastoma

**Fig. 6:**
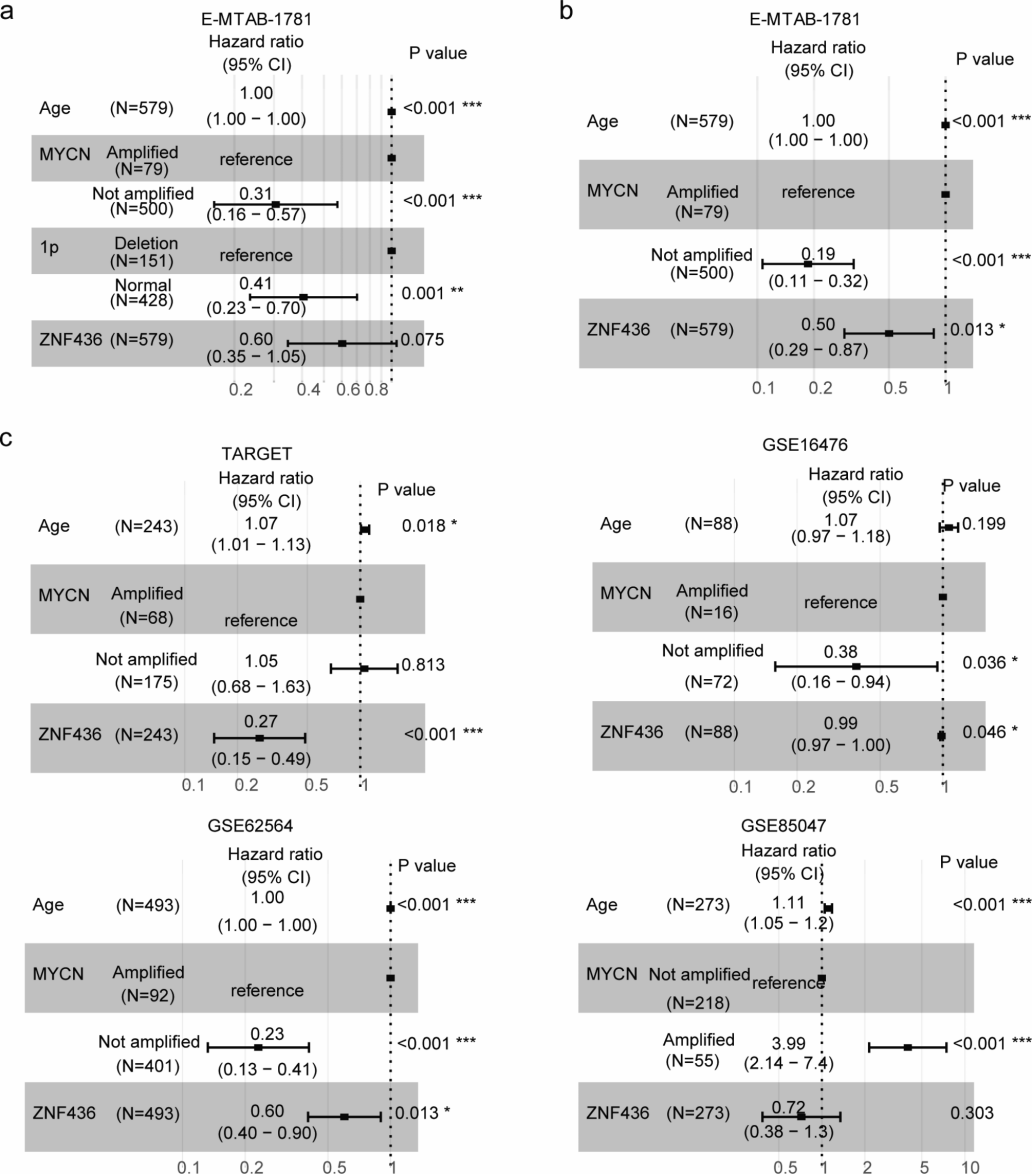
ZNF436 is an independent prognostic factor of neuroblastoma. (**a**) Forest plots showed the associations of age, MYCN amplification, 1p deletion and ZNF436 with the overall survival of neuroblastoma in E-MTAB-1781 dataset. Hazard ratio and P values were determined by multivariate cox regression assay. (**b**) Forest plots showed the associations of age, MYCN amplification and ZNF436 with the clinical overall survival of neuroblastoma in E-MTAB-1781 dataset. (**c**) The associations of age, MYCN amplification and ZNF436 with the overall survival of neuroblastoma in TARGET, GSE16476, GSE62564 and GSE85047 datasets

The associations of age of diagnosis, MYCN amplification, 1p deletion and ZNF436 expression in the prediction of the overall survival of neuroblastoma were further analyzed using multivariate cox regression assay. Age of diagnosis, MYCN amplification and 1p deletion were independent prognostic factors of neuroblastoma, while, ZNF436 was not an independent prognostic factor of neuroblastoma in E-MTAB-1781 dataset (Fig. 6a). However, ZNF436 was a prognostic factor of neuroblastoma in E-MTAB-1781 dataset independent of age of diagnosis and MYCN amplification (Fig. 6b).

Furthermore, age of diagnosis was an independent prognostic factor in TARGET, GSE62564 and GSE85047 datasets, while not in GSE16476 dataset (Fig. 6c). MYCN amplification was also an independent prognostic factor in GSE16476, GSE62564 and GSE85047 datasets, but not in TARGET dataset (Fig. 6c). ZNF436 was an independent prognostic factor of neuroblastoma in TARGET, GSE16476 and GSE62564 neuroblastoma cohorts (Fig. 6c).

### Synergistic prognostic effects of ZNF436 with MYCN amplification or age of diagnosis in neuroblastoma

**Fig. 7:**
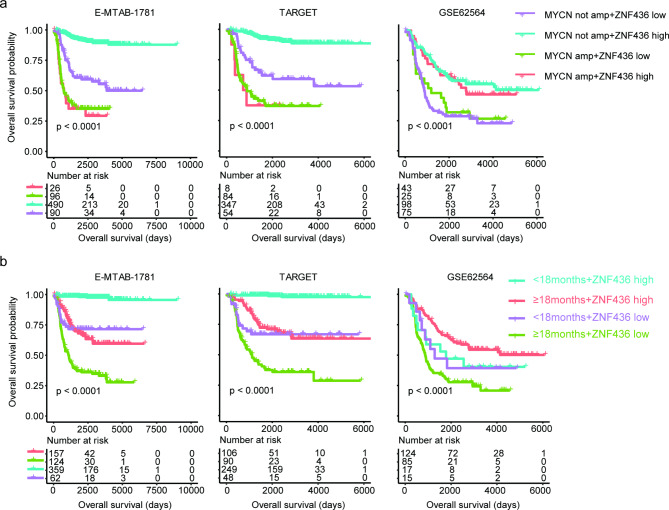
Synergistic prognostic effects of ZNF436 with MYCN amplification or age of diagnosis in neuroblastoma. (**a**) Neuroblastoma patients were classified into different sub-groups based on ZNF436 and MYCN amplification. Overall survival of each sub-group was determined using the Kaplan-Meier survival analysis in E-MTAB-1781, TARGET and GSE62564 datasets. P values were generated using log-rank test. (**b**) Neuroblastoma patients were classified into different sub-groups based on the ZNF436 and age of diagnosis. Overall survival of each sub-group was determined in E-MTAB-1781, TARGET and GSE62564 datasets

Since, age of diagnosis, MYCN amplification and ZNF436 expression were independent prognostic factors in most neuroblastoma cohorts, we speculated the superior prognostic effects in the combinations of ZNF436 with MYCN amplification or age of diagnosis. Based on MYCN amplification and ZNF436 expression level, neuroblastoma patients were classified into different sub-groups. Neuroblastoma patients with ZNF436 high expressions and without MYCN amplification had significantly longer overall survival in E-MTAB-1781 and TARGET datasets (Fig. 7a). On the contrary, neuroblastoma patients with ZNF436 low expressions and with MYCN amplification had significantly shorter overall survival in E-MTAB-1781 and TARGET datasets (Fig. 7a). However, the synergistic prognostic effects of ZNF436 with MYCN amplification in GSE62564 dataset were not significant (Fig. 7a).

Neuroblastoma patients with age at diagnosis ≥ 18months and with ZNF436 lower expressions had the worst prognosis in E-MTAB-1781, TARGET and GSE62564 datasets (Fig. 7b). On the contrary, neuroblastoma patients with age at diagnosis < 18months and with ZNF43 higher expressions had the best prognosis in E-MTAB-1781 and TARGET datasets (Fig. 7b). Neuroblastoma patients with age at diagnosis < 18months and with ZNF43 higher expressions had the medium clinical survival in E-MTAB-1781, TARGET and GSE62564 datasets (Fig. 7b). Also, the synergistic prognostic effects of ZNF436 with age at diagnosis in GSE62564 dataset were not significant (Fig. 7b). Those results highlighted the complex of neuroblastoma cohorts from different datasets and some results from one dataset could not be validated by other datasets.

### Construction of nomogram model to predict the overall survival of neuroblastoma

**Fig. 8:**
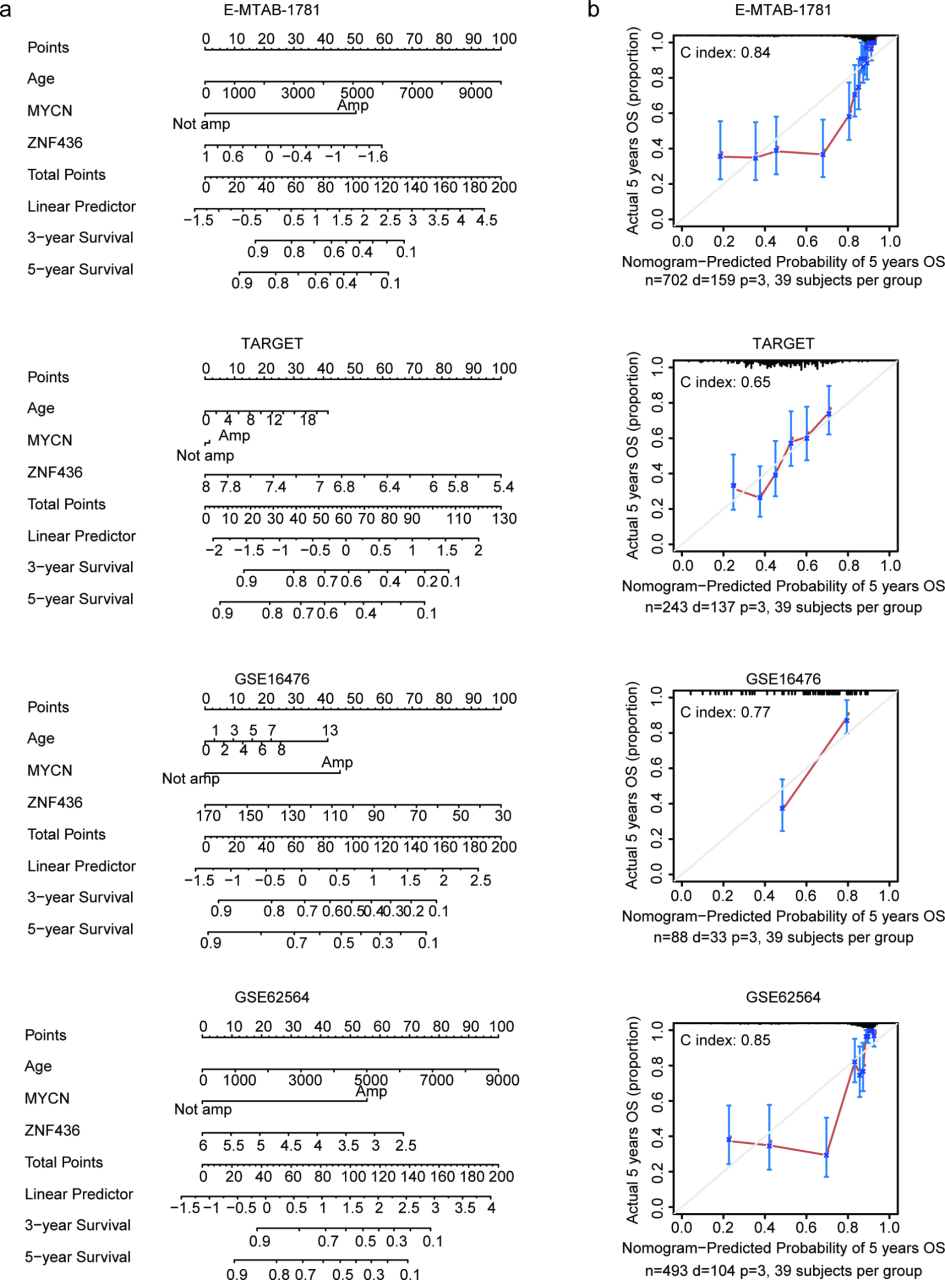
Construction of nomogram risk model based on age, MYCN amplification and ZNF436. (**a**) The nomogram models based on age, MYCN amplification and ZNF436 in TARGET, GSE16476, GSE62564 and GSE85047 datasets. (**b**) The calibration diagrams showed the accuracy of the nomogram models as evaluated using C index in TARGET, GSE16476, GSE62564 and GSE85047 datasets

Our previous results suggested that ZNF436 was associated with the overall survival of neuroblastoma and showed the synergistic prognostic effects of ZNF436 with MYCN amplification or age of diagnosis in neuroblastoma. We then constructed a nomogram model based on age of diagnosis, MYCN amplification and ZNF436 to predict the overall survival of neuroblastoma in E-MTAB-1781, TARGET, GSE16476 and GSE62564 datasets (Fig. 8a). The accuracy of the nomogram model was further evaluated using calibration diagram. The C index values indicated the high accuracy of the nomogram models (Fig. 8b).

**Fig. 9:**
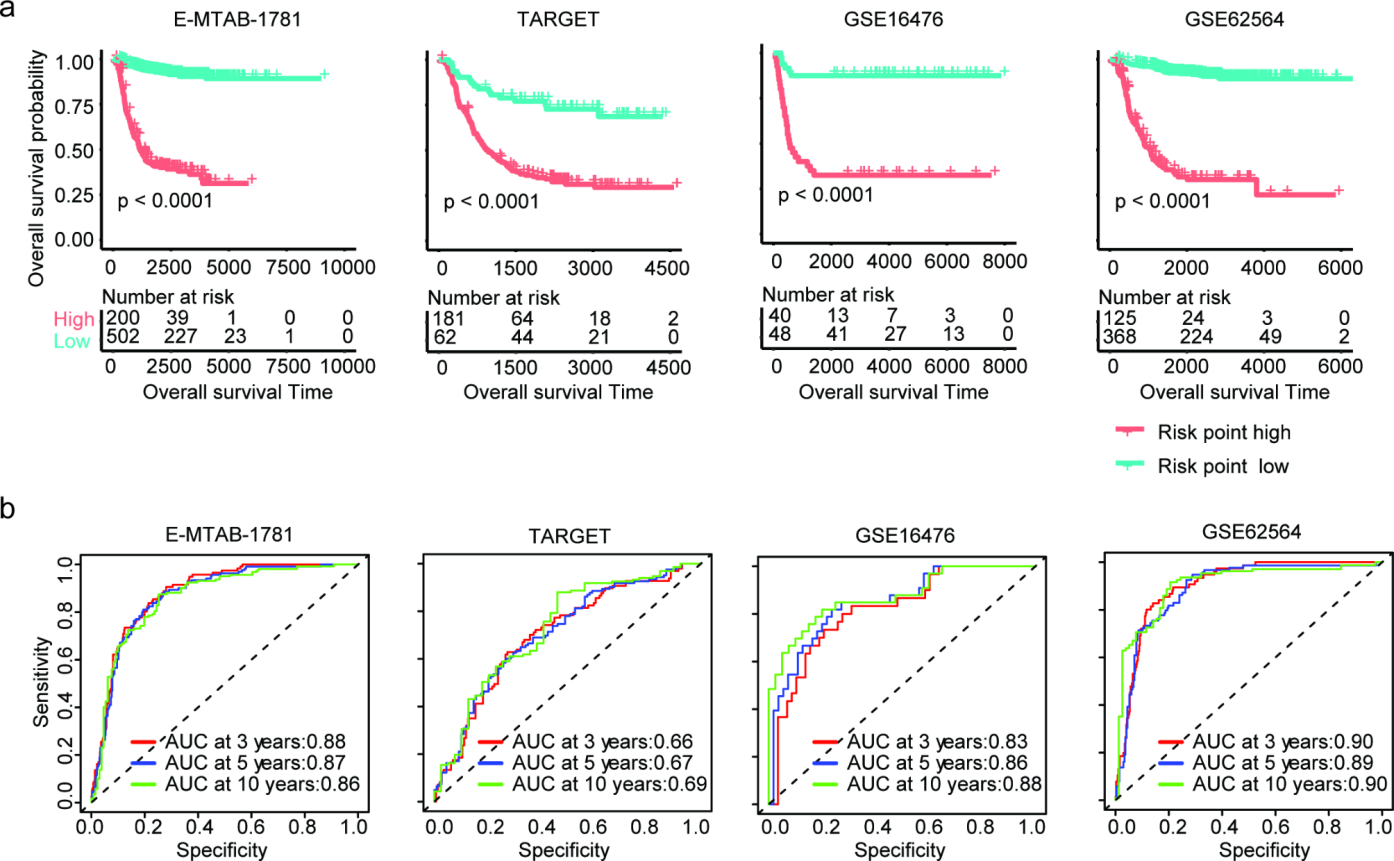
Predictive accuracy of the nomogram model. (**a**) Different overall survival of neuroblastoma with the low risk and high risk in TARGET, GSE16476, GSE62564 and GSE85047 datasets. (**b**) The ROC curves showed the predictive specificity and sensitivity of the risk points. AUC was calculated in the prediction of the overall survival of neuroblastoma in TARGET, GSE16476, GSE62564 and GSE85047 datasets

The risk point of each neuroblastoma patient in E-MTAB-1781, TARGET, GSE16476 and GSE62564 datasets was calculated from the nomogram models. Neuroblastoma with lower risk points had significantly longer overall survival than neuroblastoma with higher risk points in E-MTAB-1781, TARGET, GSE16476 and GSE62564 datasets (Fig. 9a). Moreover, in E-MTAB-1781, TARGET, GSE16476 and GSE62564 datasets, the risk points could predict the three years, five years or ten years overall survival of neuroblastoma with high specificity and sensitivity (Fig. 9b). More importantly, the predictive significance the nomogram model was higher than simple ZFN436 expression. For example, the five years overall survival prediction by ZFN436 expression was 0.76 (Fig. 5a), while, the five years overall survival prediction by the nomogram model was 0.87 in E-MTAB-1781 dataset (Fig. 9b).

## Discussion

Genetic copy number variations, particularly MYCN amplification and 1p deletion, are implicated in the progress of neuroblastoma. The amplified or deleted DNA regions can be as large as 1 Mb and include multiple suspected neuroblastoma driver or suppressor genes [39, 40]. Determining the driver or suppressor roles of correlated genes could provide more prognostic makers and potential therapeutic targets for neuroblastoma. Previously, we had analyzed the MYCN regulated genes [41], E2F regulated genes [42] and TP53 regulated genes [43] for the prognosis of neuroblastoma. Using similar datasets, in this study, we analyzed the genetic loss of 1p, and investigated the expressions and prognosis of 1p candidate genes in neuroblastoma.

Contrast with gain of 2p24.3 MYCN regions, deletions of 1p regions influenced more genes. Based on TARGET dataset, 773 genes in chromosomal 1p regions were deleted in neuroblastoma. Some genes at this chromosomal regions are associated with low risks of neuroblastoma [26]. However, not all 1p candidate genes are down-regulated with the deletion of 1p. Compared with 1p normal neuroblastoma patients, only 105 genes in 1p regions were differentially expressed in 1p deleted neuroblastoma in E-MTAB-1781 dataset. And only 11 genes were commonly changed in 1p deleted neuroblastoma patients in E-MTAB-1781, GSE13136 and GSE73517 datasets. Only ZNF436 was involved in the prognosis of neuroblastoma in all E-MTAB-1781, TARGET, GSE16476, GSE62564 and GSE85047 datasets.

ZNF436 is a transcription factor belonging to the zinc finger protein family and modulates genes expressions through binding to the DNA elements [44]. The functions of ZNF436 in neuroblastoma are never reported. In glioma, ZNF436 could promote tumor cell proliferation [45]. In breast cancer, high ZNF436 expression is associated with high metastasis [46]. Our results provided new prognostic functions of ZNF436 in neuroblastoma and those prognostic functions may be associated with unique MYCN amplification and 1p deletion. ZNF436 is located in the chromosomal 1p36.12 regions. Consistent with the poor prognosis of 1p36 deletion, lower expression of ZNF436 was associated with worse clinical outcomes of neuroblastoma. Moreover, the expression levels of ZNF436 were lower in neuroblastoma with MYCN amplification or age at diagnosis ≥ 18months, or in stage 4 neuroblastoma. ZNF436 had robust predictive values of MYCN amplification and overall survival of neuroblastoma. Furthermore, the prognostic significance of ZNF436 in neuroblastoma was independent of MYCN amplification and age of diagnosis. Combinations of ZNF436 expression with MYCN amplification or age of diagnosis achieved better prognosis of neuroblastoma.

Nomogram is extensively used in the prognosis of cancers [47–49] as well as obstetric diseases [50–53] and achieves accurate predictions. However, predictive nomogram models for the risks of neuroblastoma are unclear. So, in this study, using independent risk factors age of diagnosis, MYCN amplification and ZNF436, we developed a nomogram model to predict the overall survival of neuroblastoma. Compared with simple ZFN436 expression, the nomogram model could predict the overall survival of neuroblastoma with higher specificity and sensitivity.

To our best knowledge, this is the first integrated analysis of the expressions and prognosis of 1p candidate genes in neuroblastoma. Our results suggested that ZNF436 was served as prognostic biomarker of neuroblastoma. However, those results were generated using published neuroblastoma cohorts and lacked of additional in *vitro* and in *vivo* validations. Therefore, the detailed roles of ZNF436 should be further revealed using neuroblastoma cells and neuroblastoma patients.

## Conclusions

ZNF436 was down-regulated in 1p deleted neuroblastoma and ZNF436 was lower in neuroblastoma patients with MYCN amplification or age at diagnosis ≥ 18months, or with stage 4 neuroblastoma. Higher ZNF436 expression was associated with the longer event free survival and overall survival of neuroblastoma. ZNF436 had robust predictive values of MYCN amplification and overall survival of neuroblastoma. A nomogram risk model based on age, MYCN amplification and ZNF436 could predict the overall survival of neuroblastoma with higher specificity and sensitivity.

## Data Availability

The datasets generated and/or analysed during the current study are available from the TARGET (https://ocg.cancer.gov/), EMBL-EBI (https://www.ebi.ac.uk/arrayexpress/) and the GEO websites (www.ncbi.nlm.nih.gov/geo).

## Abbreviations

TARGET: Therapeutically Applicable Research to Generate Effective Treatments
EMBL-EBI: The European Molecular Biology Laboratory-European Bioinformatics Institute
GEO: Gene Expression Omnibus
ZNF436: Zinc finger protein 436
HR: Hazard ratio
ROC: Receiver operating characteristic
AUC: Area under the ROC curve
